# Predecisional information search adaptively reduces three types of uncertainty

**DOI:** 10.1073/pnas.2311714121

**Published:** 2024-11-15

**Authors:** Mikhail S. Spektor, Dirk U. Wulff

**Affiliations:** ^a^College of Arts and Sciences, VinUniversity, Hanoi, Vietnam; ^b^Department of Psychology, University of Warwick, Coventry CV4 7AL, United Kingdom; ^c^Center for Adaptive Rationality, Max Planck Institute for Human Development, Berlin 14195, Germany; ^d^Faculty of Psychology, University of Basel, Basel 4055, Switzerland

**Keywords:** information search, risky choices, sampling paradigm, process tracing

## Abstract

How do people search for information when they are given the opportunity to freely explore their options? Previous research has suggested that people focus on reducing uncertainty before making a decision, but it remains unclear how exactly they do so and whether they do so consistently. We present an analysis of over 1,000,000 information-search decisions made by over 2,500 individuals in a decisions-from-experience setting that cleanly separates information search from choice. Using a data-driven approach supported by a formal measurement framework, we examine how people allocate samples to options and how they decide to terminate search. Three major insights emerge. First, predecisional information search has at least three drivers that can be interpreted as reducing three types of uncertainty: structural, estimation, and computational. Second, the selection of these drivers of information search is adaptive, sequential, and guided by environmental knowledge that integrates prior expectations, task instructions, and personal experiences. Third, predecisional information search exhibits substantial interindividual heterogeneity, with individuals recruiting different drivers of information search. Together, these insights suggest that human information search is complex in ways that cannot be fully explained by monolithic accounts of information search, including proposals focused on estimation uncertainty or cost–benefit analysis. We conclude that broader theories of human information-search behavior are necessary.

## Significance

How do people search for information before making choices? Theoretical approaches suggest that decision makers’ primary aim is reducing the uncertainty surrounding aspects such as an option’s average value; however, this view has rarely been tested using empirical data. This article reports a data-driven analysis of a large-scale, publicly available dataset aiming to identify the drivers of information search. It reveals that people use their prior expectations and environmental knowledge to adaptively reduce at least three types of uncertainty: structural uncertainty concerning features of the decision environment, estimation uncertainty surrounding options’ average rewards, and computational uncertainty arising from the difficulty of information processing. The findings highlight how information search is more complex and adaptive than portrayed by current theoretical accounts.

Information search is an integral part of the decision-making process. Every choice we make is based on information that must first be obtained (1). In many cases, information search is inherently consequential. For example, in trying out a new restaurant, we forgo the opportunity to eat at our favorite place around the corner. How people navigate this balance between maximizing short-term gains and obtaining new information that can be useful in the long run has been thoroughly investigated (2, 3). However, information search is not always consequential. Modern information technology is creating ever-more opportunities to freely explore the available options before coming to a consequential decision: We can consult online restaurant reviews (4, 5), use internet platforms to study the development of financial investments (6, 7), or use simulations to learn about the dangers of diseases (8) before making a decision. What drives information search in these situations?

The current empirical evidence suggests that nonconsequential information search, where information can be obtained without making a choice, is driven by a process that weighs the expected gains from additional information against the potential costs associated with acquiring it (9–13). It has been observed that people search more when information is of higher value [e.g., when incentives are larger (14, 15) or outcome variability is higher (16)] and search less when information comes at a higher cost [e.g., when direct costs are incurred in the form of fees or indirect costs result from missing out on the opportunity to maximize payoff (17)]. How humans implement the trade-off between the benefits and costs of acquiring information is, however, poorly understood.

Theoretical accounts of nonconsequential information search tend to focus on the idea that the benefit of search is uncertainty reduction (11, 12, 18, 19). Most take a narrow view by equating “uncertainty” with “estimation error” in terms of the discrepancy between the belief about the value of an option and its actual value. This error can arise due to various factors, such as incomplete information or variability in the observed information (19). However, competing views consider broader notions of uncertainty, including higher-order uncertainty about the structure of the decision environment (20). Not knowing about key environmental features, such as the typical range or number of outcomes, limits people’s ability to estimate decision-relevant criteria. Crucially, qualitatively different kinds of search behavior may be needed to reduce estimation uncertainty and structural uncertainty. Whereas estimation uncertainty can generally be reduced by sampling from the option with greater estimation error, this approach will not necessarily reduce structural uncertainty. Additionally, whether these two are the only types of uncertainty considered during information search and how important they are remains unclear. It is not even clear whether (any kind of) uncertainty reduction is the primary driver of nonconsequential information search: People might also, for example, sample from options with higher average rewards, irrespective of the uncertainty involved (21).

Even less is known about the “cost” side of information search in terms of how and when people decide to terminate search. One proposal is that information search is guided by an evidence-accumulation process that terminates search when a given evidence threshold has been reached (11, 18, 22–26). Another is that decision makers have an inherent propensity to terminate search and that this propensity is adjusted based on the experience of surprise or volatility (19). However, there has been scant comparison of these or other possible proposals. Again, other drivers entirely may be involved. People could decide in advance how much time they want to spend on information search, as has been observed in animal foraging (27), or stop searching when specific conditions are met, such as having observed certain patterns (28) or experienced all possible outcomes (11, Appendix F). Notably, such strategies can be understood as reducing a third type of uncertainty, “computational uncertainty,” which concerns potential errors in the computations involved in search or choice. A reduction of computational uncertainty can be achieved by simplifying the search process or the integration of information—for example, by aiming for easy denominators to simplify the calculation of relative frequencies.

Previous studies of nonconsequential information search have also largely disregarded the possibility that a single search is subject to multiple drivers. Yet work using process tracing methodology suggests that search consists of qualitatively distinct phases, with early phases being directed at discovering the overall structure of the choice environment rather than estimating specific properties (29, 30). Furthermore, search might be influenced by prior expectations and knowledge about the decision environment with respect to aspects such as the range and number of outcomes to be experienced. Studies manipulating the amount of structural information provided through experimental instructions have revealed changes in search behavior that support the idea that individuals’ expectations and knowledge are crucial in shaping how they search (10, 31, 32). These observations suggest that decision makers’ actual approach to nonconsequential information search may well differ from the predictions of current theoretical accounts (33–35). Ultimately, there may be multiple drivers of information search, and people may differ in the drivers they recruit, consistent with individual differences in visual search and learning mechanisms (36–39).

## Search in the Sampling Paradigm of Decisions from Experience.

**Fig. 1.**
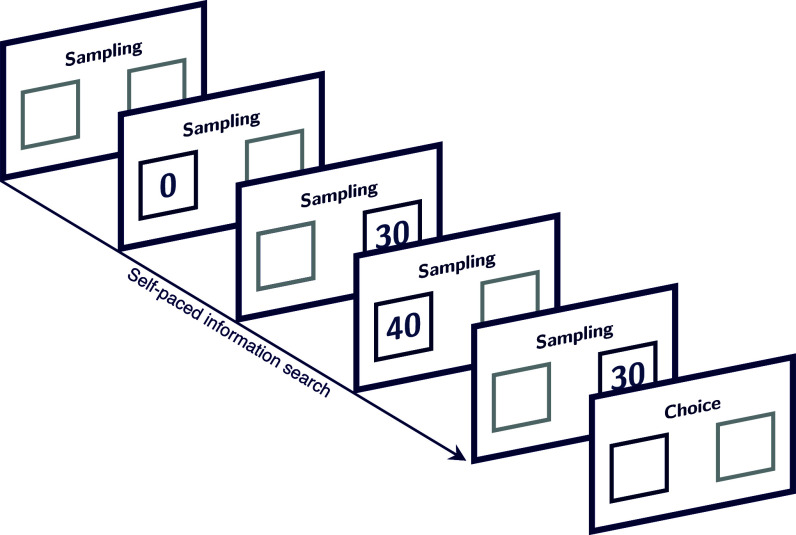
Illustration of the sampling paradigm. Individuals search for information at their own pace, sampling freely from the options’ outcome distributions until they decide to make a single consequential choice.

To identify the drivers of nonconsequential information search, we analyzed a large, publicly available database comprising over 1,000,000 information-search decisions made by over 2,500 individuals within the sampling paradigm of decisions from experience (see *Materials and Methods* for details). In this paradigm, participants choose between two monetary lotteries (options). Each option is characterized by an outcome distribution (i.e., a random variable whose realizations are monetary values) that is initially unknown to participants. Before making a choice, they can obtain information about each of options at no cost by sampling (i.e., observing individual realizations from the respective outcome distributions) for as long as and in whatever order they like. Once participants are satisfied that they have obtained enough information, they terminate information search by indicating that they want to make a choice. Subsequently, they make a single consequential and typically incentivized decision (see Fig. 1 for an illustration). The sampling paradigm is distinct from other decision-from-experience paradigms, including many reinforcement-learning tasks (40), in that information search and choice are cleanly separated. Other decision-from-experience paradigms involve consequential search, in which people can only obtain information about an option’s outcome distribution by making a consequential choice and obtaining the chosen option’s reward or punishment. As search in the sampling paradigm does not require real choices, it is well suited for studying the principles underlying predecisional information search (23).

The database comprises 55 sampling-paradigm studies that vary in several respects, including the participants’ cultural backgrounds, the wording of the instructions, and the mechanics of sampling (e.g., the input device or the delay between samples). Crucially, the studies also vary in the kinds of decision problems presented. On average, participants were presented with 15.16 decision problems (range: 1 to 131, SD = 19.16) involving pairs of lotteries. Most studies (*N*= 47) featured binary lotteries with at most two outcomes, but four studies featured lotteries with up to three outcomes and two studies featured lotteries with up to five outcomes. Two distinct types of decision can also be distinguished: Risky–safe problems pair a risky option (i.e., an option with more than one unique outcome) with a safe option offering the same outcome each time, whereas risky–risky problems pair two risky options.

By analyzing information search in the sampling paradigm, we aim to help elucidate the drivers of information search in an important area of human psychology and to complement work investigating information search in other domains, such as memory (41–43), physical space (44, 45), and the internet (46, 47). Previous work has suggested that a common cognitive underpinning for search across environments regulates aspects such as how humans navigate the trade-off between different drivers of search (48, 49). Consequently, we expect that analyzing this large database of human information-search behavior will also help advance the understanding of information search more generally. Our analysis follows a data-driven approach using a formal measurement framework of information search, which allows different drivers of information search to be specified under the same computational umbrella. We seek to address two fundamental questions. First, how do people allocate samples during information search—in other words, how do they decide which option to sample from? Second, how and when do people decide to terminate information acquisition and make a consequential choice? Our findings show that predecisional information search has several drivers related to the reduction of at least three different types of uncertainty.

## Results

### Measurement Framework.

We used a computational measurement framework to assess the importance of different drivers of information-search behavior. This measurement framework assumes that people ultimately seek to identify and choose the option with the higher average reward. This assumption is consistent with work showing a strong preference for the option with the higher average experienced outcome (11, 22, 23, 25, 26) and several theoretical accounts of nonconsequential information search in decisions-from-experience paradigms (18, 19). It is assumed that people estimate the mean and variance of each option using a Bayesian updating process. This process holds prior beliefs about the options’ properties and adjusts those beliefs based on the information sampled.

The central equation of our framework, presented in detail in *Materials and Methods*, is the posterior distribution of the average reward $\mu_{i}$ for option i given its observed outcomes $y_{i}$:

$$p\left(\mu_{i}|y_{i}\right)=t_{\nu_{n,i}}\left(\mu_{n,i},\frac{\sigma_{n,i}^{2}}{\kappa_{n,i}}+\delta_{i}\right)$$

It states that the average reward follows a shifted and scaled t-distribution, with expected value $\mu_{n,i}$ and variance $\left(\frac{\sigma_{n,i}^{2}}{\kappa_{n,i}}+\delta_{i}\right)\frac{\nu_{n,i}}{\nu_{n,i}-2}$.

The derivation of this equation is based on the assumption of normally distributed outcomes, which is technically at odds with the categorical outcome distributions of the lotteries analyzed. The motivation underlying our measurement framework is not to construct a cognitive process model but to derive latent entities that reflect distinct drivers of information search and are able to take participants’ prior expectations into account. Moreover, the model represents a pragmatic and plausible approximation to the cognitive process.

#### Drivers of information search.

By assessing the options people sample from, our measurement framework allows us to quantify the importance of three drivers of information search: estimation-driven search, discovery-driven search, and value-driven search.

Two of these drivers concern the distribution’s variance or, in other words, the uncertainty in the estimation of the average reward. The variance, defined as $\frac{\sigma_{n,i}^{2}}{\kappa_{n,i}}+\delta_{i}$, consists of two components, which reflect two central types of uncertainty that can drive information search. The first component, which consists of the quotient of the option’s variance ($\sigma_{n,i}^{2}$) and the parameter $\kappa_{n,i}$, which is directly related to sample size, reflects estimation uncertainty. The estimation uncertainty surrounding an option’s average reward can thus be reduced by taking more samples, especially from the option with the larger estimation uncertainty. We call sampling from the option with the larger estimation uncertainty estimation-driven search. The second component, which is captured by the parameter $\delta_{i}$, reflects structural uncertainty related to the number of unique outcomes that an option yields. The parameter is a binary variable whose value is extremely high ($10^{10}$) when there still are unique outcomes that have not yet been observed and zero when all possible unique outcomes have been observed. This parameter, which extends the standard formalization of posterior variance, is included to capture a second driver of information search, namely, to discover missing unique outcomes. This will eventually lead to observation of the missing unique outcome and thereby reduce the combined uncertainty by $\delta_{i}$, which will drop out of the equation, leaving only estimation uncertainty. We call sampling from the option with higher combined uncertainty discovery-driven search. Note that discovery-driven search is reduced to estimation-driven search once all unique outcomes have been observed. A third driver of search postulated in past work is captured by the mean of the posterior around the average reward. We call sampling from the option with the higher posterior mean value-driven search.

#### Accounting for prior expectations.

Another crucial feature of our measurement framework is that it can take into account people’s expectations through the use of informed priors. We use an informed prior that depends on the variance in the expected values of the options encountered by a participant in a given study. This prior is intended to reflect the realistic scenario that participants have a somewhat accurate expectation of where the average rewards in a given study will fall. For the first decision problem, people can draw inferences based on, for example, the projected duration of the experiment or the expected bonus payment; for later decision problems, they have collected first-hand experience of the typical range of outcomes.

Prior expectations are typically neglected in computational models of information search, but they can substantially affect the framework’s predictions by determining how the model behaves in situations of limited information. The priors for the average reward play a particularly important role. When the sample average differs substantially from the prior for the average reward, this results in high uncertainty. Generally speaking, the sample average will deviate strongly from the prior when unique outcomes are still missing, which makes informed priors naturally sensitive to structural uncertainty. One important consequence of this is that estimation-driven search can mimic discovery-driven search when informed priors are used. To show the crucial role of priors and demonstrate this (partial) mimicry, we also present results using a noninformative prior, where the posterior is determined solely on the basis of the observed samples.

### How Are Samples Allocated during Information Search?

**Fig. 2.**
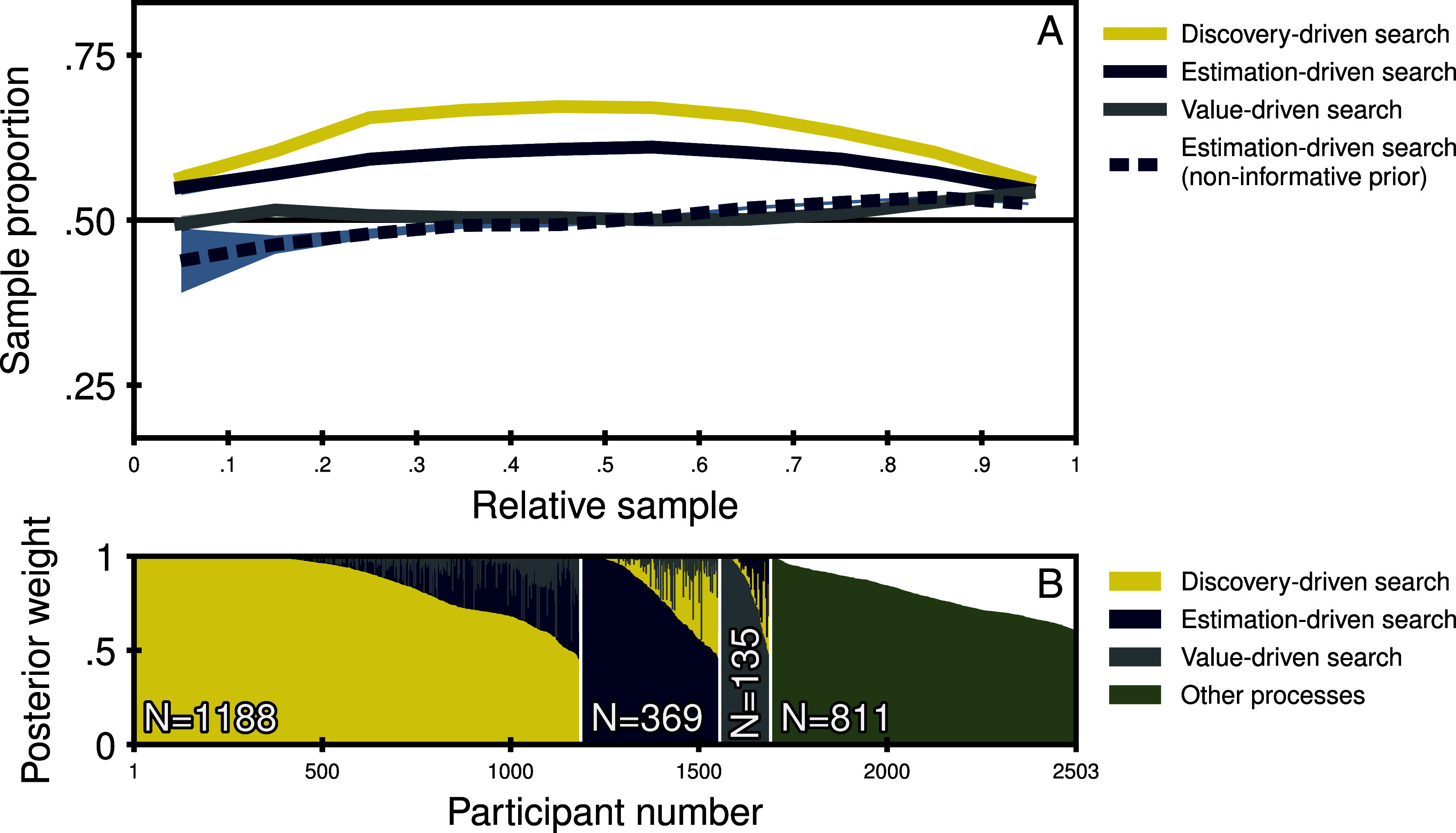
Drivers of information-search allocation at the aggregate and individual level. Panel (*A*) shows the proportion of sample-allocation decisions consistent with value-driven search (blue), estimation-driven search (gray), and discovery-driven search (yellow) as a function of the position of a sample within the sampling sequence (relative sample). All three model-based measures were aggregated across all participants and problems. Shaded areas indicate the 95% CI. Panel (*B*) shows the posterior weights for each individual and driver of information search. The x-axis is separated into four groups, depending on the predominant driver. Within each group, individuals are sorted in descending order by the posterior weight of that driver. The last group consists of individuals who were not described by any of the drivers above chance level. Here, the heights of the bars reflect the posterior mean of the error parameter.

The first question we address is which drivers underlie the allocation of samples to options during search. To this end, we first used our measurement framework to determine the predictions for value-driven search, estimation-driven search, and discovery-driven search, which is possible for each trial after participants have sampled at least once from each option. We then quantified how often participants’ sampling aligned with each driver’s predictions. Fig. 2*A* shows the proportion of sampling decisions in line with each driver as a function of the relative position within the sampling sequence. It reveals that very few choices were in line with value-driven search, as reflected in only minor deviations from the 50% line (51.5%) that corresponds to random behavior. This is in contrast to findings reported previously (21, 50). The only systematic deviation from randomness for value-driven search appears to occur at the very end of the sampling sequence, where there seems to be a slight systematic tendency to sample from the option with the higher posterior mean (see Fig. 2*A*, blue line). However, in light of people’s tendency to choose the option with the higher posterior mean (74.1% of all choices), this late increase is likely a by-product of optional-stopping behavior, which leads people to sample from the option they are about to choose just before terminating information search. This is a common phenomenon in information-search behavior (e.g., refs. 22 and 42) and has been dubbed a gaze-cascade-like effect in decisions from experience in reference to gaze-cascade effects in visual search (51).

People’s search seemed instead to be estimation-driven and discovery-driven, with overall proportions of 58.6% (95% bootstrapped CI: [58.5%, 58.7%]) and 62.9% (95% bootstrapped CI: [62.8%, 63.0%]), respectively. Both made identical predictions in 82.6% of the sampling decisions. This is no surprise given that the two processes are nested within one another, with discovery-driven search reducing to estimation-driven search as soon as all unique outcomes have been observed. Considering only the cases where the two drivers make distinct predictions, we found that sampling decisions aligned more with discovery-driven search (62.4%). Overall, therefore, discovery-driven search provides the best account of how people allocate samples in nonconsequential information search.

To further investigate the prevalence of discovery-driven search, we conducted a classification analysis at the level of individuals (see Fig. 2*B* and *Materials and Methods* for details), evaluating the extent to which individuals made information-search choices consistent with each of the three drivers. Overall, 47.5% (95% bootstrapped CI: [47.3%, 47.8%]) of participants were found to primarily engage in discovery-driven search, 14.7% (95% bootstrapped CI: [14.7%, 15.1%]) in estimation-driven search, and 5.4% (95% bootstrapped CI: [5.3%, 5.5%]) in value-driven search. The remaining 32.4% (95% bootstrapped CI: [32.0%, 32.4%]) were classified as not consistent with any of the three drivers. All in all, these results suggest that the discovery of missing unique outcomes and, more generally, the reduction of structural uncertainty is dominant during sampling allocation, with other drivers—particularly reduction of estimation uncertainty—playing a significant role for at least some individuals and situations.

### The Role of Epistemic States in Uncertainty-Driven Search.

The dominance of discovery-driven search suggests that people’s primary motivation when sampling is to discover all missing unique outcomes. But how can they tell when further unique outcomes are still to be discovered? We seek to answer this question by analyzing people’s sampling allocation as a function of epistemic states that differ in whether or not any additional unique outcomes can be observed. These situations arise from two key properties of the data that they share with most research on risky choices. First, options typically contain few discrete outcomes (e.g., winning $4 with a probability of 80% or otherwise nothing); second, the choice problems encompass two types of choice situations, either a choice between a risky and a safe option (i.e., a single sure outcome) or a choice between two risky options. These properties create epistemic states that are equivalent in terms of the number of unique outcomes observed but differ in whether additional unique outcomes can be observed or not. For example, people who have observed two unique outcomes from one option and one unique outcome from another (a state we refer to as “2–1”) may be facing a risky–safe problem, implying that no additional unique outcomes are to be discovered, or a risky–risky problem, implying that one additional unique outcome is still to be discovered (assuming the typical case of a maximum of two outcomes per option). This pattern extends to situations with more unique outcomes. For example, people’s knowledge can also be incomplete when they have seen three unique outcomes from one option and two unique outcomes from the other (a state we refer to as “3–2”) when the maximum number of unique outcomes is three. We analyzed people’s sampling allocations in a total of five epistemic states, including three states of complete structural information (“2–1” in risky–safe problems, “2–2” in risky–risky problems with two unique outcomes per option, and “3–3” in risky–risky problems with three unique outcomes per option) and two states of incomplete structural information (“2–1” in risky–risky problems with two unique outcomes per option and “3–2” in risky–risky problems with three unique outcomes per option). Note that our database included other states, but they were either far less frequent or not informative of specific search strategies (e.g., “1–1”). If people know that additional unique outcomes are yet to be observed, this should be reflected in the proportion of allocations consistent with discovery-driven search.

**Fig. 3.**
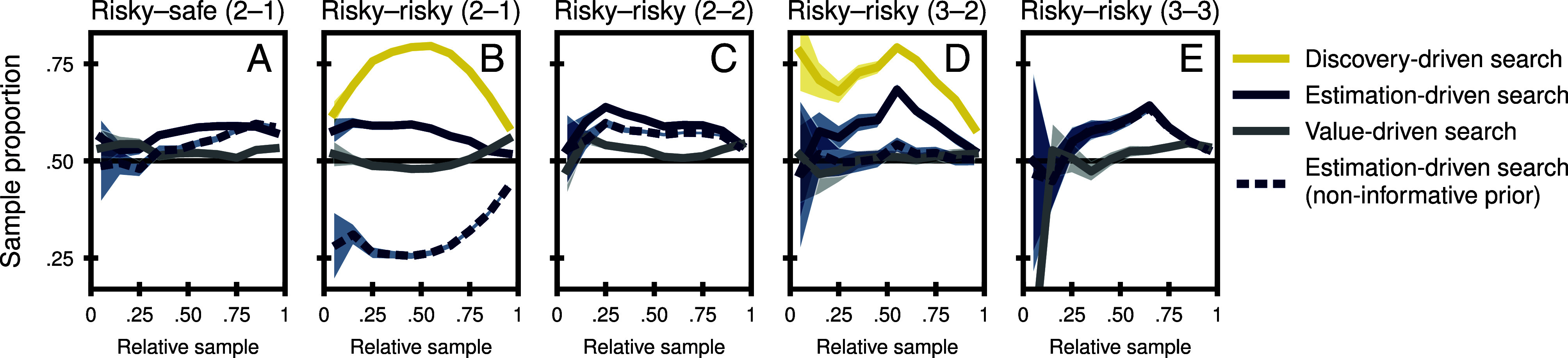
Sampling allocation and epistemic states. The figure shows engagement in value-driven search (blue), estimation-driven search (gray), and discovery-driven search (yellow) as a function of the position of a sample within the sampling sequence (relative sample) and the epistemic state. Panel (*A*) shows situations with one two-outcome risky option (i.e., an option with two possible outcomes) and one safe option (i.e., an option that has only one possible outcome) and in which all possible outcomes have been observed (i.e., two from the risky option and one from the safe option; denoted 2–1). Panel (*B*) shows situations with two two-outcome risky options and in which participants have observed both outcomes from one option but only one outcome from the other (denoted 2–1). Panel (*C*) shows the same decision situation as (*B*) but after all possible outcomes have been observed (denoted 2–2). Panel (*D*) shows situations with two risky options that have three outcomes each and in which participants have observed all three outcomes from one option but only two outcomes from the other (denoted 3–2). Panel (*E*) shows the same decision situation as (*D*) but after all possible outcomes have been observed (denoted 3–3). Solid lines are derived from a model with an informed prior; dashed lines are derived from a model with noninformative priors. Shaded areas indicate the 95% CI.

Fig. 3 presents the results of this state-specific allocation analysis. People in the 2–1 state of a risky–safe problem (panel *A*) showed a small but systematic preference for estimation-driven search, which—in this case of complete experience—is identical to discovery-driven search. However, participants in the 2–1 state of a two-outcome risky–risky problem (panel *B*) overwhelmingly preferred discovery-driven search (73.5%) over estimation-driven search (56.5%). As soon as they reached the complete 2–2 state (panel *C*), they again preferred estimation-driven search, similar to the patterns observed in the 2–1 state of risky–safe problems. The same pattern of results can be observed as people transition from the 3–2 state of three-outcome risky–risky problems (panel *D*; 59.5% estimation-driven search vs. 70.3% discovery-driven search) to the complete experience 3–3 state (panel *E*). These results show that the drivers of sample allocation are adjusted to the environmental situation: When people are in a situation of incomplete experience, with one more unique outcome left to be observed, they engage in discovery-driven search, presumably in order to discover the missing outcome; when they are in a situation of complete experience after having observed the previously missing unique outcome, they switch to estimation-driven search.

### Mechanisms of Inferring the Structure of the Decision Environment.

The results of our state-specific analysis of allocation decisions suggest that people know when additional unique outcomes are to be discovered, implying knowledge of the structure of the decision environment. Specifically, they seem to know whether they are in a risky–safe or a risky–risky environment and how many unique outcomes the risky options have. While this may seem puzzlingly clairvoyant, there are at least three possible mechanisms by which people can infer the environmental structure of a problem.

#### The role of experimental instructions.

The instructions that participants receive before starting a task can provide information about the structure of decision problems—for example, by communicating the range or number of unique outcomes. Past work has shown that manipulating the instructions given on decision problems can affect people’s choices (32). We used a subset of our database to analyze the impact of such instructions: The same experiment was presented to two groups of participants, with only one being informed about the number of outcomes they could expect (52). We predicted that participants with prior information about the number of unique outcomes would more frequently engage in discovery-driven search in incomplete states of experience than those without such prior information and that this effect would be especially pronounced in the first few problems encountered, when participants’ first-hand environmental knowledge was still limited. In line with this prediction, across the first three problems, participants with prior information selected the option consistent with discovery-driven search more frequently than those without prior information (difference of 7.7 percentage points). Across all 60 problems, in contrast, there was practically no difference between the groups (0.4 percentage points). This finding suggests that instructions about the number of unique outcomes might play some role in triggering discovery-driven search. Unfortunately, because the instructions for most studies in our database are unavailable, we cannot test their effect more widely. Moreover, a large portion of the studies (50.9%) used problems of risky–risky and risky–safe types, where information about the problem structure would be of limited use.

#### The role of problem-by-problem experience.

People may learn about the structure of the environment from their experience in the study (53, 54). As mentioned, studies usually use an uneven mix of risky–risky or risky–safe problems or, in some cases, only one of these types, implying that participants can, in principle, infer the likely type of environment from previously encountered problems. We evaluated this possibility by analyzing how people responded to the first experience of a fourth unique outcome, which is unambiguous evidence that the environment can be risky–risky. We expected these participants to engage in more discovery-driven search in the 2–1 state, where they could reasonably expect one more outcome to be discovered. Our analysis was constrained in several ways. It only considered studies 1) that included risk–risky problems, 2) in which participants experienced a fourth unique outcome, 3) in which participants encountered at least one problem in which they experienced three unique outcomes before observing a fourth unique outcome for the first time, 4) in which participants subsequently faced at least one additional problem, and 5) that contained information about the order of problems. We identified 154 individuals from 17 studies who satisfied all five conditions and evaluated whether their engagement in discovery-driven search in the 2–1 state was affected by the experience of a fourth unique outcome. This was indeed the case. The proportion of allocations aligned with discovery-driven search rose from 55% to 66% (Cohen’s d=0.39,95%CI=[0.22,0.55]). Moreover, when we focused the analysis on the problems immediately before and after the experience of a fourth unique outcome, the difference increased from 11 percentage points to 24 percentage points (49% vs. 73%, Cohen’s d=0.46,95%CI=[0.30,0.63]). These results suggest that prior experience explains some portion of people’s situation-specific discovery-driven search.

#### The role of perceived difficulty.

Finally, people may infer the existence of discoverable unique outcomes from the difficulty of distinguishing between the two options at any given point in time. In most studies, decision problems are designed to be difficult, offering a choice between two options with nearly indistinguishable expected values. However, this difficulty emerges only when all possible unique outcomes have been observed. Consider the popular problem presenting a choice between one option offering $32 with a probability of 2.5% (or $0 otherwise) and another option offering $3 with a probability of 25% (or $0 otherwise). With full experience, the expected values are $3.2 and $3, respectively. However, missing any unique outcome results in much stronger differences, effectively trivializing the choice. As a result, people can infer that further unique outcomes remain to be discovered if the current situation seems too easy to be true.

An alternative mechanism that can mimic this type of high-level inference is baked into the informed prior of our model. With informed priors, the estimation uncertainty depends on the difference between the observed sample averages and the prior mean, which is likely to be high when unique outcomes are still missing. As a result, the estimation uncertainty for the option with a missing unique outcome will be larger, leading to the allocation of more samples to that option, even in the absence of discovery motives. The influence of this prior-based process can be revealed by replacing the informed prior underlying estimation-driven search with a noninformative prior and contrasting the results. Panel *A* of Fig. 2 shows the results for uncertainty-driven search under the noninformative prior as a dashed line. As can be seen, the proportion of estimation-driven sample allocations drops by 6.8 percentage points under the noninformative prior (to 51.7%). This decrease was primarily driven by situations of incomplete information (reduction by 24.5 percentage points and 8.2 percentage points, see Fig. 3 *B* and *D*, respectively). In situations of complete information, the change in priors had no meaningful impact (Fig. 3*C* and *E*). Note that using a noninformative prior would also harm discovery-driven search. However, as discovery-driven search is fully determined by the number of missing unique outcomes in each option, performance drops only from 62.9% (informed prior) to 59.2% (noninformative prior).

The difference between the two specifications for estimation-driven search underscores the power of prior expectations and environmental knowledge for sample allocation. However, as the change in priors does not fully close the gap between estimation- and discovery-driven search, people’s hunt for missing unique outcomes is still better accounted for by discovery-driven search. This result suggests that people track aspects of their environment beyond the average outcome. Specifically, they may track the maximum number of unique outcomes of an option and decide to allocate samples to options with missing unique outcomes.

### Summary.

To summarize, participants’ allocation of samples to options was mostly in line with discovery-driven search, reflecting a motivation to discover missing unique outcomes. In contrast, participants never quite relied on value-driven search, and it was only when no further unique outcomes remained to be discovered that sample allocation was consistently in line with estimation-driven search (Fig. 3 *C* and *E*). Search seems to be guided by knowledge about the structure of the environment, which can derive from experimental instructions, past experience, and assessments of the difficulty of problems. Overall, participants explored their environment in a strategic manner, switching adaptively between discovery-driven and estimation-driven search and relying on prior expectations and environmental knowledge.

### How Is Information Search Terminated?

The second question, we seek to address is how people decide to terminate information search. Past work suggests that people stop collecting additional samples when the level of evidence is high or when the level of surprise is low (11, 18, 19, 55). Within our measurement framework, both proposals can roughly be conceptualized as stopping when the pooled posterior uncertainty surrounding the options’ means is at its lowest point. We analyzed this for the two main types of uncertainty-driven search, namely estimation-driven and discovery-driven search. We found that people stopped their search when the uncertainty underlying estimation-driven search was at its lowest in 79.4% of cases and when the uncertainty underlying discovery-driven search was at its lowest in 90.6% of cases. These results give credence to proposals focusing on evidence and surprise but also suggest that people consider whether missing outcomes remain to be discovered when deciding to terminate search.

We further investigated the relationship between the discovery of unobserved unique outcomes and search termination in two ways. First, we analyzed the number of samples drawn before and after the final missing unique outcome was discovered to detect whether people stopped immediately after discovering the final outcome. To simplify the analysis, we focused on problems in which each option had two unique outcomes (i.e., two-outcome risky–risky environments), which make up the majority of the database. Overall, people took considerably more samples after discovering the fourth and final unique outcome (38% of all samples) than between discovering the second and third unique outcome (19%); however, in 17.7% of cases, this relationship was reversed. This suggests that most people continued to explore after experiencing the last unique outcome.

**Fig. 4.**
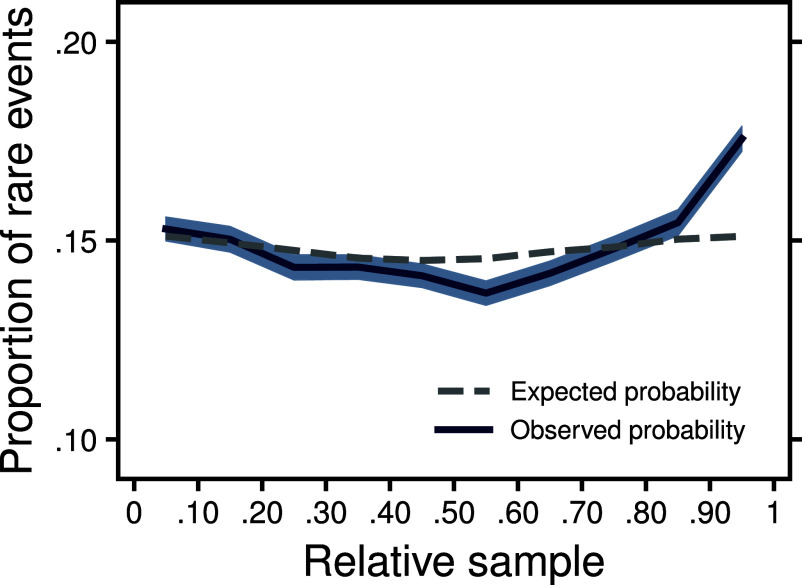
Probability of observing the outcome with the lowest probability (rare event) depending on the position of a sample within a sampling sequence (relative sample). The gray line depicts the expected probability of rare events occurring given their true probabilities and the empirical sample sizes. The blue line depicts empirical observations of rare events and the shaded area depicts the 95% bootstrapped CI.

Our second analysis focused on the relative frequency of observing the rare event, the event with the lowest probability across both options, as a function of its position in the sampling sequence. If at least some people rely primarily on structural uncertainty to terminate search, an amplification of the frequency of rare events toward the end of the sampling sequence can be expected: In most cases, rare events are the final outcome to be discovered because they are the least likely to occur. Stopping straight after the rare event has been observed will increase the experienced relative frequency of observing the rare event—analogous to optional stopping in data collection that increases the rate of type-1 errors (56). Fig. 4 shows the proportion of rare events (blue line) across the sampling sequence relative to a baseline of experiencing the rare event under deterministic stopping based on simulations using the empirical sample size distribution (dashed gray line). The results show a clear increase in the proportion of observing the rare event toward the end of the sampling sequence. This result again highlights that structural uncertainty plays an important role in search termination. In consequence, rare events are experienced more often than they should be according to their probability, and people who apply discovery-driven termination will likely obtain a biased view of decision problems.

### Signals of Fixed Search Termination.

We also investigated another search termination strategy: fixed search. Findings suggest that people sometimes stop search after taking a predetermined number of samples, independently of what they have observed (27, 28), which can help simplify the computations underlying search and choice and thus reduce computational uncertainty.

**Fig. 5.**
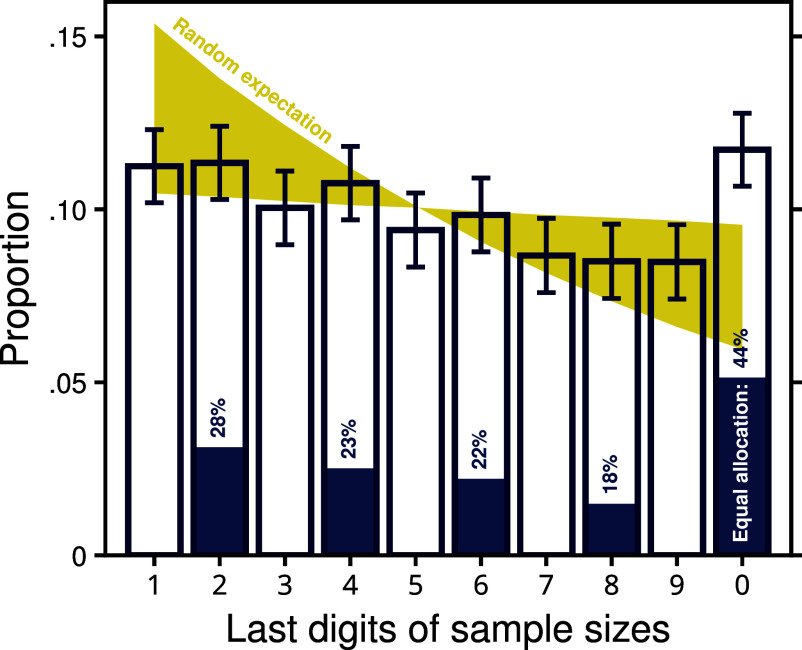
Distribution of last digits of the sample sizes drawn. The yellow shaded area represents the baseline expectation under the assumption that each person has a fixed probability between 1% and 10% of terminating information search after each sample. The hollow bars represent the distribution of observed last digits. The blue bars represent the proportion of problems for which the samples were equally allocated between the two available options (heights relative to the hollow bars). Error bars indicate the 95% CI.

We investigated this possibility using an analysis inspired by Benford’s law (57), which states that in many naturally occurring datasets, the leading digit is more likely to be small than large. In our case, we analyzed the last digit of all sample sizes of 11 or above, which, in most cases, was the second-leading digit. Our motivation was to detect unusually frequent sample sizes. To obtain a baseline reflecting the random expectation, we assumed that each participant had a constant probability (varying between 1% and 10%) of terminating search after each sample, and we simulated search termination for each problem in the database. As shown in Fig. 5 (yellow shaded area), under a fixed probability of terminating information search, stopping was consistent with Benford’s law: Sample sizes ending with a “1” were, on average, twice as likely as sample sizes ending with a “0.” The last digits of the empirical sample sizes also tended to obey this trend, but with two important exceptions (Fig. 5): First, odd last digits were, on average, less frequent than the next even digits, although fixed probability stopping predicts the opposite. Second, and more importantly, the last digit “0” not only countered the trend but was the most frequent last digit altogether (11.7%), followed by “2” (11.3%). These results demonstrate that people have a tendency to stop on sample sizes that are even and, particularly, divisible by ten. It thus appears that some searches were terminated when participants had reached a predetermined sample size.

To further corroborate the existence of fixed search termination, we analyzed how often people allocated samples evenly across the options. The proportion of problems with even allocation (displayed as blue shaded areas in Fig. 5) is strongly elevated when the sample size ends in “0,” at 43.8%, which is higher than either the average of the other even sample sizes (23.1%) or the expected even allocation rate based on a binomial distribution of samples under random allocation (14.6%). Based on these results, it seems probable that some people recruit a specific search strategy consisting of allocating half of a predetermined sample size to each of the two options.

### Summary.

To summarize, our analysis of sampling termination supports the idea that people terminate search in different ways. Minimization of estimation uncertainty appeared to be the dominant objective, as indicated by extensive search even after all unique outcomes had been experienced. However, discovery-driven search termination also played an important role, as reflected in two observations. First, people stopped most frequently when the combined uncertainty as implemented in discovery-driven search was at its lowest. Second, rare events were experienced disproportionally frequently toward the end of a search sequence. Both observations suggest that some people terminated search immediately following complete experience. Finally, some people appeared to take a different search-termination approach by stopping after taking a predetermined sample size and allocating samples evenly across the two options, potentially in an attempt to minimize computational uncertainty. Overall, these findings confirm that multiple drivers underlie human information search.

## Discussion

How do people search for information when they can do so freely? The present study aimed to clarify the drivers of nonconsequential information search by investigating a dataset of over 1,000,000 sampling decisions made within the sampling paradigm in a decision-from-experience setting. Converging evidence from a Bayesian measurement framework and data-driven analyses supports three major observations. First, sample allocation and search termination have multiple drivers, involving at least three types of uncertainty: estimation, structural, and computational uncertainty. Second, information search is guided adaptively by individuals’ prior expectations and knowledge of the environment. Third, individuals differ in the information search drivers they recruit. Together, these observations illuminate the complex cognitive machinery underlying human information search and provide empirical findings for future formal models of search.

Our analyses produced evidence that information search addresses different types of uncertainty. People’s search begins with a focus on reducing structural uncertainty by seeking out unobserved unique outcomes. Some people terminate search immediately after discovering the final unique outcome, resulting in a characteristic overoccurrence of rare events toward the end of their search sequence, which biases their experiences. However, most people continue to search by shifting their focus to reducing estimation uncertainty until they reach a global minimum. We further observed a tendency to allocate samples evenly and to terminate search at round sample sizes, which likely reflects a fixed-search strategy. This strategy can be understood as reducing a third type of uncertainty, namely computational uncertainty. By sampling round numbers and evenly allocating samples across options, people simplify the computations involved in sampling and determining the options’ average rewards: Sample sizes divisible by ten provide an easy denominator for calculating relative frequencies, and even allocation of samples can render denominators irrelevant as only the sums of outcomes need to be computed.

Our analysis also showed that people engage in search that is informed by their prior expectations and environmental knowledge. People seem to transition from reducing structural uncertainty (via discovery-driven search) to reducing estimation uncertainty as soon as the former has been achieved. It is highly likely that people accumulate knowledge about the structure of the task environment by integrating prior expectations, study instructions, and observed outcomes, and that they use this knowledge and experience to inform their subsequent information search. One possible algorithmic implementation of such knowledge acquisition is given in the prior of our measurement framework, which reflects the idea that people learn about the options typically encountered and their values. However, given that this prior did not fully close the gap between estimation- and discovery-driven search, it seems that people’s environmental knowledge extends beyond the estimation of values and is also likely to include the number and kinds of outcomes to expect.

Ultimately, our results demonstrate that predecisional information search unfolds in an intricate manner. Although our results are consistent with optimizing the trade-off between costs and benefits (42) or being resource-rational (58), they also suggest that human information search does not follow from a monolithic mechanism. Future research needs to take into account that predecisional information search may have multiple drivers that reduce different types of uncertainty (20) and that operate in sequence. Similar observations have been made in research using process-tracing technology to study search in other paradigms, namely, that people might initially search to orient themselves and only later transition to evaluating choice options more closely (29, 59–62). More generally, future research needs to seriously consider the mental models that people use to make sense of the task and that play an important role in guiding information search (31, 32). Crucially, these models can differ substantially from experimenters’ assumptions, which may result in the misinterpretation of human search behavior (22, 33–35, 63).

### Limitations and Future Directions.

Some limitations of our study warrant discussion. First, by affording nonconsequential information search, the sampling paradigm mimics many but by no means all real-life decision situations involving search. Whether and to what extent our findings generalize to other kinds of predecisional information search remains an open question. For example, do our findings hold when search is consequential, as in the various kinds of reinforcement-learning tasks (e.g., ref. 64)? Reasons to assume that our findings will translate include research proposing a common cognitive foundation underlying all information search (48), as well as the close links between the different learning paradigms. However, the consequential nature of obtaining information will likely affect the mechanistic nature of information search. We hope that future work will take up the challenge of investigating the drivers of information search as well as the environmental dependencies uncovered in our analyses using other paradigms.

A second and related limitation is that the database we used relies almost exclusively on highly stylized choices between monetary lotteries with discrete outcomes. In this respect, the sampling paradigm differs little from other value-based learning paradigms such as probability learning or reinforcement learning (64, 65). Nevertheless, it is important to realize that these situations do not capture the richness and multimodality of naturally occurring search environments. Future work should study situations that are more representative of everyday decision-making (8).

Third, although our analyses are based on a formal measurement framework, our findings are motivated by an exploratory, data-driven approach. Due to the large size of the database and the diversity of studies included, we are confident that our results are robust. Still, it is important to bear in mind that most of the studies included were designed to test hypotheses related to choice rather than information search. As such, they may not represent the most informative or encompassing testing ground for investigations of information search. Future studies should specifically target the drivers identified in our analyses and their dependencies on environmental knowledge. This could help provide insights into how the drivers correlate with each other and with known choice phenomena such as risk aversion or the weighting of rare events (22). A good starting point would be to assess more decision problems per participant than were typically available in our database.

Fourth, and finally, although we highlight many results immediately relevant to theory building and propose a computational framework that is able to characterize the various drivers of information search, we do not detail a complete process-level account of information search, including how individuals form environmental knowledge and what kind of structural information they attend to (e.g., sequential dependencies between outcomes or whether outcomes depend on choices). Such a model would far exceed the level of complexity of contemporary information-search models and would require many parametric and auxiliary assumptions. We believe that model building can sometimes stand in the way of fully appreciating the complexity of behavior. One example is research on reinforcement learning, which has a nearly century-long tradition of formal modeling but only recently begun to consider uncertainty minimization—let alone minimization of structural or other types of uncertainty—as a search driver. We observe that empirical paradigms and the accompanying model development have been overly reliant on the traditional model classes that have been used for decades (66). While we of course welcome theoretical developments accounting for the findings presented here, we also see merit in presenting patterns that are not predicted by current theoretical accounts without necessarily detailing a new one.

To conclude, the present work highlights the need for analyses that go beyond idealized notions of information search and instead look at how people actually approach the task at hand (35). Much contemporary decision-making research is based on the idea of independent, identically distributed observations; this approach seems to forget that people enter the experiment with expectations and work through multiple trials, giving them the opportunity to learn, to update their behavior, and to form expectations about the experimenters’ hypotheses. Part of the problem is that most research has failed to take structural uncertainty and other drivers of search seriously, instead assuming monolithic mechanisms and using normative or otherwise researcher-generated models of the environment as the lens through which to interpret human behavior (20, 66–68). Taking a broader approach to information search can reveal how search is subject to different drivers and how people integrate knowledge across the entire experiment, allowing them to act adaptively and dynamically. There is a growing body of work showing adaptive behavior in related domains (69–71). We hope that future efforts to model and study information search will account for the diversity of behavior and thought that ensues from people’s creative approaches to information search.

## Materials and Methods

### Database.

Our analysis draws on the raw data from a recent meta-analysis of the description–experience gap: Wulff et al. (22) obtained the data from 28 papers reporting on 80 datasets in which 4,400 individuals made a total of 45,239 decisions in the sampling paradigm. In each trial, participants decided between two lotteries with initially unknown outcome distributions. In the sampling phase, they could draw nonconsequential samples from the lotteries’ outcome distributions. They then committed to a single consequential choice. We restricted our analysis to data from participants who sampled autonomously (i.e., where samples were generated randomly from the true underlying outcome distributions and participants could decide when to terminate search; 89% of all decisions) and sampled at least once from each option (99.4% of all decisions). An additional three decisions were excluded due to incomplete data. After applying these exclusions, the final dataset consisted of 2,638 individuals in 55 datasets (23 papers), who made a total of 40,081 decisions and drew a total of 1,065,753 samples.

These participants faced 22,959 decisions (57%) in which both lotteries had positive or zero outcomes; 11,913 decisions (30%) in which both had negative or zero outcomes; and 5,209 decisions (13%) with mixed outcomes. In most of the decisions (29,417; 73%), both options had at least two outcomes (risky vs. risky); in 10,321 decisions (26%), one option had a single, sure outcome (risky vs. safe); and in 343 decisions (1%), both options had a single, sure outcome (safe vs. safe). In trials where one of the two options had a higher expected value (EV), participants tended to choose the higher EV option more often than not (22,528 out of 38,201 decisions; 59%). With control for sampling error, participants maximized (i.e., chose the option observed to have the better outcomes on average: the higher experienced mean) in 74% of trials (29,063 out of 39,249 decisions). In trials where one of the two options was de facto riskier (in terms of the true underlying variance of outcomes), participants tended to avoid the riskier option (16,974 choices of the riskier option in 39,052 decisions; 43%). On average, participants drew 26.59 samples (range: 2 to 750, SD = 24.80, median = 20). Most of the time, participants sampled several times from one option before switching to the other. The switching rate (i.e., number of switches relative to number of samples after the first sample) covered the whole possible range (0 to 1, M = 0.30, SD = 0.37, median = 0.10).

For our analyses, we used all relevant data and excluded individual samples only where it was not possible to compute the dependent variable—for example, when participants only sampled from one option. For the analyses involving sampling dynamics within a single problem, we used relative samples in order to control for differences in absolute sample sizes. Relative samples anchor the first and last samples to 0 and 1, respectively, and linearly project all samples between these two poles. For example, if an individual drew five samples, then the relative samples corresponding to sample numbers 1, 2, 3, 4, and 5 would be 0, 0.25, 0.5, 0.75, and 1, respectively.

Bootstrapped CIs were obtained via 1,000 samples of nonparametric bootstrapping within participants and problems.

### Measurement Framework.

According to our computational framework, people are Bayesian observers who hold prior beliefs about the properties of the outcome distributions underlying the available choice options and update these beliefs with incoming information (i.e., with the observed samples). The framework further assumes that decision makers want to identify and select the option with the higher average mean; this assumption is generally consistent with previous work (11, 18, 22–26). As an auxiliary assumption, our computational framework posits that samples stem from a univariate normal distribution with an unknown mean and variance. This assumption is crucial for simplifying the measurement framework and is psychologically and statistically reasonable given people’s strong parametric bias toward normal distributions (73, 74) and the central limit theorem.

Our measurement framework follows the normal-distributed data model with a conjugate prior, as derived by Gelman et al. (75). Here, the joint posterior distribution of the mean $\mu_{i}$ and variance $\sigma_{i}^{2}$ of the to-be-estimated normal distribution of option i after observing the data $y_{i}$ follows a normal-inverse-$\chi^{2}$ distribution:

$$p\left(\mu_{i},\sigma_{i}^{2}|y_{i}\right)=\;\text{N-Inv-}\chi^{2}\left(\mu_{n,i},\frac{\sigma_{n,i}^{2}}{\kappa_{n,i}}+\delta_{i};\nu_{n,i},\sigma_{n,i}^{2}\right),$$

where n in the subscript refers to the posterior beliefs. The parameters of the posterior distribution correspond to the location ($\mu_{n,i}$) and the scale ($\frac{\sigma_{n,i}^{2}}{\kappa_{n,i}}+\delta_{i}$) of $\mu_{i}$ and the degrees of freedom ($\nu_{n,i}$) and scale ($\sigma_{n,i}^{2}$) of $\sigma_{i}^{2}$. Parameter $\delta_{i}$ reflects the completeness of the experience, in terms of having observed all unique outcomes across both options, assuming a value of $10^{10}$ when the experience is incomplete and 0 when it is complete. To clarify, we assume that decision makers are not interested in the dispersion of the data but only in estimating the expected value of the distribution. The marginal posterior distribution of the expectation, $\mu_{i}$, follows a shifted and scaled t distribution with $\nu_{n,i}$ degrees of freedom, location parameter $\mu_{n,i}$, and scaling parameter $\frac{\sigma_{n,i}^{2}}{\kappa_{n,i}}+\delta_{i}$:

$$p\left(\mu_{i}|y_{i}\right)=t_{\nu_{n,i}}\left(\mu_{n,i},\frac{\sigma_{n,i}^{2}}{\kappa_{n,i}}+\delta_{i}\right)$$

Parameters are updated as a function of the prior beliefs, denoted by o in the subscript, the mean $\bar{y_{i}}$ and variance $s_{i}^{2}$ of the observed samples, and the number of observations $n_{i}$:

$$\begin{array}{cc} \mu_{n,i} & =\frac{\kappa_{o,i}\mu_{o,i}+n_{i}\bar{y_{i}}}{\kappa_{o,i}+n_{i}}\\ \kappa_{n_{i}} & =\kappa_{o,i}+n_{i}\\ \nu_{n_{i}} & =\nu_{o,i}+n_{i}\\ \nu_{n_{i}}\sigma_{n,i}^{2} & =\nu_{o,i}\sigma_{o,i}^{2}+\left(n_{i}-1\right)s^{2}+\frac{{\left(\bar{y_{i}}-\mu_{o,i}\right)}^{2}\kappa_{o,i}n_{i}}{\kappa_{o,i}+n_{i}} \end{array}$$

For derivations and proofs of the analytical expressions, see ref. 75, pages 67 to 68.

The main parameters of interest of this model are $\mu_{n,i}$, the posterior expectation of the value of the outcome distribution, and the uncertainty surrounding the posterior mean, defined as $\left(\frac{\sigma_{n,i}^{2}}{\kappa_{n,i}}+\delta_{i}\right)\times \frac{\nu_{n,i}}{\nu_{n,i}-2}$ for $\nu_{n,i}>2$.

Based on these two expressions, we determine model-based markers of value-driven search, estimation-driven search, and discovery-driven search for every sample. The marker of value-driven search is selecting the option with the higher expectation ($\mu_{n,i}$). The marker of estimation-driven search is selecting the option with higher uncertainty considering only the component of variance that relates to estimation uncertainty, namely $\frac{\sigma_{n,i}^{2}}{\kappa_{n,i}}$. Finally, the marker of discovery-driven search is selecting the option with higher uncertainty considering the full posterior variance, including the $\delta_{i}$ parameter.

The priors of the model were set as follows. Except for specific analyses highlighted in the main text, informed priors were used for $\mu_{o,i}$ and $\sigma_{o,i}^{2}$. These priors were set to the mean and the variance, respectively, of the expected value of all options across all problems that a participant encountered in an experiment. Note that this implies a slight variation in priors across participants. If a participant encountered only one problem in a study, the distribution of expected values within the study was used instead. We set the precision parameter $\kappa_{o,i}$ to 1 and the degrees-of-freedom parameter $\nu_{o,i}$ to 2. The latter represents the smallest possible value that permits an analysis of all data. Values smaller than 2 imply loss of observations due to the $\nu_{n,i}-2$ denominator in the uncertainty formula. For some analyses, we also used a noninformative prior. In this case, we set all parameters to 0, except the prior $\nu_{o,i}=-1$, so the posterior degrees of freedom corresponded to n−1.

### Search Classification Model.

We used a hierarchical Bayesian classification model inspired by previous work (76) for the analysis of individual differences. The general idea is that the sample-allocation decisions people make are governed by imperfect implementations of the three drivers of information search, namely estimation-driven search, discovery-driven search, and value-driven search. The model assumes two main differences across individuals captured by two parameters: a) the degree to which they rely on each of the drivers, which is captured by a decision weight parameter, and b) the implementation error rates of the drivers, which is captured by an error parameter.

Formally, the sampling decision $y_{i,t}$ of person i on trial t is determined by a mixture of three processes k∈K:

$$y_{i,t}∼\sum_{k=1}^{K=3}\omega_{i,k}\left(\left(1-\epsilon_{i}\right){\hat{y}}_{k,t}+\epsilon_{i}\right)$$

Here, ${\hat{y}}_{k,t}$ denotes the (deterministic) predictions of process k (discovery-driven search, estimation-driven search, and value-driven search) obtained within our measurement framework under informed priors. The parameter $0\leq \epsilon_{i}\leq 1$ reflects an individual-specific implementation error according to which the option predicted by the driver is chosen with probability $\left(1-\epsilon_{i}\right)$ and one of the two available options is chosen at random with probability $\epsilon_{i}$. When ϵ=0, people’s sample allocations are perfectly predicted by some mixture of the three drivers; when ϵ=1, none of them are. Finally, parameter $\omega_{i,k}$ is the mixture weight that reflects the probability with which an individual relies on each of the three drivers, where $\sum \omega_{i,k}=1$.

The hierarchical structure of the model is as follows. A person’s vector of mixture weights $\vec{\omega_{i}}$ comes from a group-level Dirichlet distribution with the concentration vector $\vec{\alpha }>0$: $\vec{\omega_{i}}∼Dir\left(\vec{\alpha }\right)$. A person’s trembling-hand error $\epsilon_{i}$ is a transformation of raw errors on the unconstrained parameter space $\epsilon_{r,i}$ that is drawn from a normal distribution with mean μ and SD σ: $\epsilon_{r,i}∼N\left(\mu ,\sigma \right)$. The raw errors on the unconstrained parameter space are then transformed to the [0,1] space using a Φ transformation: $\epsilon_{i}=\Phi \left(\epsilon_{r,i}\right)$, where Φ is the cumulative distribution function of the standard normal distribution $N\left(0,1\right)$.

We used weakly informative prior distributions for the group-level parameters. For the concentration vector of the group-level Dirichlet distribution, we used a zero-centered half-Cauchy distribution with a scaling parameter γ=3. For the normal distribution, we used a N(0,1) distribution for the mean, resulting in a uniform $U\left(0,1\right)$ of the average implementation-error rate across individuals. For the SD, we used a half-normal HN(0,1) distribution.

To obtain samples from the posterior distribution of the classification model, we used a no-U-turn sampler as implemented in Stan (77). We ran four randomly initialized chains in parallel for at least 7,500 total iterations, of which the first 2,500 were discarded as warm-up samples. We then thinned the remaining 5,000 iterations per chain by a factor of 20, so that 250 samples remained per chain for a total of 1,000 posterior samples from all four chains. We confirmed convergence using the $\hat{R}$ statistic (ref. 75, p. 285), with all $\hat{R}\leq 1.012$, reflecting very good convergence.

We used a two-step procedure to identify the search drivers that most often accounted for each participant’s search. In the first step, we used the widely applicable information criterion (78, 79) to exclude participants whose sample-allocation behavior could not be accounted for at above chance level by the three drivers of information search, while accounting for model flexibility. This was the case for N=811 individuals. For the remaining N=1,683 individuals who were well described by the model, we used Bayesian hypothesis testing via posterior exceedance probabilities (see ref. 80 for an introduction) to determine which drivers each individual recruited most frequently. A posterior exceedance probability is closely related to hypothesis testing using Bayes factors but quantifies the evidence for a hypothesis in terms of a summary statistic in a set of hypotheses rather than a ratio. For example, if the evidence in favor of a hypothesis A is nine times that of hypothesis B (i.e., ${\text{BF}}_{\mathrm{AB}}=9$), then the posterior exceedance probability of hypothesis A is $Pr\left(A\right)=\frac{{\text{BF}}_{\mathrm{AB}}}{1+{\text{BF}}_{\mathrm{AB}}}=\frac{9}{10}$. We obtained the posterior exceedance probabilities for each driver of information search and each participant by counting the proportion of posterior samples for which the respective driver had the highest weight for said participant. This approach is a straightforward generalization of the standard method to obtain directional Bayes factors (81).

We validated the results of the classification model by assessing for each of the identified clusters (as determined by the individual’s primary driver) whether their sample allocation was in line with the model’s predictions. Table 1 shows the proportion of sample allocations in line with each of the three drivers of information search as a function of cluster. The results confirm that for each of the classes, people’s samples were predominantly guided by the respective driver (in bold type).

**Table 1.** Sample allocation within clusters of participants

Cluster	Discovery	Estimation	Value
Discovery (N=1,188)	**66.0%**	58.8%	51.0%
Estimation (N=369)	61.8%	**66.9%**	52.3%
Value (N=135)	53.3%	52.6%	**62.2%**
Rest (N=811)	47.6%	47.3%	47.7%

## Data Availability

Anonymized data and the full analysis code can be found on the Open Science Framework: https://doi.org/10.17605/OSF.IO/NQ6PK (72).
